# Optimizing vermicompost-soil ratios for synergistic enhancement of *Allium fistulosum* growth dynamics and phytochemical quality

**DOI:** 10.3389/fpls.2025.1694011

**Published:** 2025-10-24

**Authors:** Shuangmei Gong, Xiulong Ou

**Affiliations:** Research Center for Preparation and Properties of New Functional Materials, Hanjiang Normal University, Shiyan, Hubei, China

**Keywords:** vermicompost, *Allium fistulosum*, optimal ratio, vitamin C, soluble sugar, nitrate

## Abstract

To determine the optimal vermicompost application ratio in vegetable cultivation, this study focused on *Allium fistulosum* (variety NC89) and conducted a pot experiment with five treatments, using vermicompost-to-soil mass ratios of 0% (control), 25%, 50%, 75%, and 100%. Growth indicators and quality parameters were measured, and the vermicompost effect was analyzed based on morphological characteristics. Results showed that under the 50% treatment, the vitamin C and soluble sugar contents of *A. fistulosum* were significantly higher than those under other treatments. The biological yield and fresh weight were relatively high, and growth indicators such as root length and plant height were well - coordinated. The number of tillers was significantly greater, with dark green leaves and compact texture. The 75% treatment had the lowest nitrate content, but its growth indicators were slightly inferior to those under the 50% treatment. A comprehensive analysis indicated that a 50% vermicompost-to-soil ratio could synergistically improve *A. fistulosum* yield and quality by enhancing soil aeration and balancing nutrient supply. This study provides a theoretical basis and technical reference for rational vermicompost application in protected vegetable cultivation.

## 1 Introduction

Vermicompost, as a product of organic waste decomposed by earthworms, is regarded as an efficient bio-organic fertilizer due to its richness in humic acid, amino acids, beneficial microorganisms, and plant growth regulators (Arancon et al., 2019). Its unique granular structure can improve soil aeration and water-holding capacity, and regulate the rate of nutrient release through microbial activity, thus offering significant advantages in reducing chemical fertilizer application and improving crop quality (Yang et al., 2024a).

After vermicompost is applied to soil, it can regulate the activity of organic nutrients to inhibit pathogen damage of pathogens to plant roots. The antagonistic microorganisms contained in it contains can also suppress the reproduction of pathogenic bacteria, enrich the soil antagonistic soil flora, and reduce soil-borne diseases (Moustafa et al., 2020; Singh et al., 2023). Microorganisms play a key role in the assimilation of organic matter by earthworms (PerezLosada et al., 2022); their activity can not only increase soil minerals and humus but also produce substances such as vitamins, antibiotics, and auxins, which promote root development, stimulate crop growth, and enhance disease resistance (ur Rehman et al., 2023; Ahmad et al., 2025). Vermicompost is also rich in various plant hormones (ur Rehman et al., 2023; Ahmad et al., 2025). The combination of auxins, cytokinins, gibberellic acid (GA), and humic acid in its extract can promote stem cutting rooting of stem cuttings (Arancon et al., 2019), and these hormones are crucial for plant metabolism. In addition, due to its high porosity and specific surface area, vermicompost is an efficient adsorbent for odors, while the large number of microorganisms it contains can also absorb and purify odorous substances (Ahmad et al., 2025).

With excellent physical, chemical, and microbial properties, vermicompost is widely used in horticulture and crop cultivation. It can improve the germination rate of various crops such as grains, legumes, flowers, and vegetables, promote growth, increase yield, and enhance quality (Kumar et al., 2019; Aechra et al., 2022; Gul and Gidik, 2023; Jiang et al., 2023; Akef Bziouech et al., 2024; Tomic et al., 2025). The development of fertilizers in China has progressed from farmyard manure to chemical fertilizers. However, long-term excessive application of chemical fertilizers, combined with insufficient input of organic fertilizers, has led to the consumption of soil organic matter depletion, declining soil fertility, reduced yield-increasing efficiency of chemical fertilizers, and a decline in the quality of agricultural products. At the same time, the rapid development of animal husbandry has made it difficult to fully utilize livestock manure, and its discharge has caused pollution, further worsening the rural ecological environment.

As a new type of high-efficiency organic fertilizer, vermicompost can create a soil environment conducive to vegetable growth and pathogen suppression. It can improve soil permeability, looseness, the abundance of beneficial flora, soil temperature, and organic matter content (Aechra et al., 2022; Mustafayeva et al., 2022; Jiang et al., 2023). It promotes growth, inhibits soil-borne diseases, and improves soil structure, thereby reducing the use of synthetic fertilizers (Mokgophi et al., 2020). Vermicompost has broad application prospects, and its research and promotion of such bio-organic fertilizers can not only help solve the problem of disposal of rural and industrial organic waste disposal (Zaman and Yaacob, 2022; Katiyar et al., 2023; ur Rehman et al., 2023) but also improve fertilizer quality and advance ecological agriculture.

In recent years, research on vermicompost application of vermicompost in vegetable cultivation has gradually increased. For example, studies by Amuza (Amuza and Ilie, 2022) showed that the extract dose of vermicompost extract has a positive impact on maize seed germination. Dah Pahlavan’s experiment on maize crops demonstrated that an appropriate vermicompost ratio can significantly increase yield and reduce nitrate accumulation (DahPahlavan et al., 2023). Jankauskienė et al. (2022) found that adding vermicompost to a peat substrate positively affects cucumber seedling physiology, and that total cucumber yield of cucumbers grown in a peat–vermicompost substrate was 7.4%–11.1% higher than that of plants grown in peat substrate alone (Jankauskienė et al., 2022).

*Allium fistulosum (Allium fistulosum ‘Caespitosum’)*, a widely cultivated leafy vegetable, has attracted considerable attention for its quality traits (such as vitamin C and soluble sugar content) and safety indicators (nitrate content) (Cepuliene et al., 2024). However, there are few studies on the effect of vermicompost on the growth of *A. fistulosum*, and most have focused on single-ratio applications, lacking systematic comparisons of different dosages. This study evaluated five vermicompost–soil mixing ratios of vermicompost and soil, systematically measured the growth indicators and quality parameters of *A. fistulosum*, and clarified the optimal application ratio through statistical analysis, providing data support for the standardized application of vermicompost in the cultivation of *Allium* vegetables.

## 2 Materials and methods

### 2.1 Instruments

Plastic flowerpots (top diameter 15 cm, bottom diameter 10.5 cm, height approximately 16 cm, thickness approximately 0.8 cm); measuring cylinders; 50 ml, 100 ml, 500 ml, 1000 ml conical flasks; test tubes, test tube racks, droppers, tweezers, scissors, spatulas, small mortars, glass rods, funnels; pipettes (1 mL, 5 mL, 10 mL) with rubber bulbs; Petri dishes (diameter 90 mm); qualitative filter paper (diameter 11 cm); vernier calipers (0.02 mm); platform balance; electronic universal furnace (model: DL-1, Beijing Yongguangming Medical Instrument Factory); water bath; asbestos mesh; desktop high-speed centrifuge (model: TGL-16C, Hunan Xingke Scientific Instrument Co., Ltd.); electronic balance (maximum 220 g, d=0.1 mg, Sartorius Scientific Instruments (Beijing) Co., Ltd.); ultrasonic cleaner; refrigerator (BCD-272/HC, Kelon brand refrigerator, Guangdong Kelon Electrical Holdings Co., Ltd.); 752 type UV–visible spectrophotometer (model 752, Shanghai Spectrum Instruments Co., Ltd.); and thermostatic blast drying oven (DHG-9076A, Shanghai Jinghong Equipment Co., Ltd.).

### 2.2 Test crop

The *Allium fistulosum* variety used was NC89. This tillering (clumping) scallion variety was developed by Shandong Agricultural University and is characterized by strong tillering ability (high yield), high stress resistance (cold, heat, and disease tolerance, disease resistance), rapid growth, and a rich flavor profile. These traits make it a preferred choice for many professional growers, particularly widely cultivated in northern China. Seeds with the same growth potential, number of tillers, uniform size, and quality, tiller number, and growth potential were selected, disinfected with ultraviolet irradiation, and air-dried. The soil for the pot experiment was collected from the suburbs of Yichang City, Hubei Province, and classified as sandy brown soil. The seedling pots were plastic trays with a top diameter of 15 cm, a bottom diameter of 10.5 cm, a height of approximately 16 cm, and a thickness of approximately 0.8 cm.

### 2.3 Test materials

Test soil: Collected from the suburbs of Yichang City (30°42′N, 111°18′E), sandy brown soil. Basic physical and chemical properties: pH 6.8, organic matter 12.5 g·kg, total nitrogen 0.82 g/kg, available phosphorus 15.6mg·kg, available potassium 98.3 mg·kg.

Vermicompost: Supplied by an earthworm farm in Hubei Province. The species *Eisenia fetida* was used, decomposing cow dung as the base material. Composition: organic matter 32.6%, humic acid 28.3%, total nitrogen 3.8%, total phosphorus 1.2%, total potassium 1.1%, pH 7.2, and beneficial bacteria content 1.2×10^8^ CFU·g.

Test crop: *Allium fistulosum* variety NC89. Seeds were soaked in 55°C warm water for 20 min and then air-dried for later use.

### 2.4 Experimental design

The experiment included five treatments, with mass ratios of vermicompost-to-soil mass ratios of 0%, 25%, 50%, 75%, and 100%. Each treatment had three replicates. Each pot contained 800 g of substrate, 15 seeds were sown, and the seedlings were thinned to 10 plants after emergence. Pots were placed at the experimental base under natural light, with an average daily temperature of 20–25°C. They were watered weekly to 70% of the field capacity every week, and weeds and pests were controlled regularly (**Table 1**).

**Table 1:** Mixing ratio of vermicompost and test soil.

Quality	0%	25%	50%	75%	100%
Vermicompost (g)	0	200	400	600	800
Test soil (g)	800	600	400	200	0

### 2.5 Determination indicators and methods

Growth indicators: After 45 days of planting, plant height, root length, and root diameter (five roots randomly measured with a vernier caliper) were recorded. After harvest, the fresh weight was measured, and plants were fixed at 105°C for 30 min, then dried at 70°C to constant weight to determine the biological yield.

Data were analyzed using SPSS 22.0 for one-way analysis of variance (ANOVA), LSD multiple comparisons (p<0.05), and correlation analysis. Figures were generated using Origin 2025.

Quality indicators: Vitamin C (VC) was determined by spectrophotometry, soluble sugar by anthrone colorimetry, and nitrate by salicylic acid colorimetry.

## 3 Experimental operations

### 3.1 Sowing

When the temperature rose to 18–25°C in mid-March, plastic flowerpots and *Allium fistulosum* seeds were taken out, exposed to sunlight for half a day, and disinfected with ultraviolet irradiation. An appropriate amount of water was sprayed on the vermicompost to ensure that its humidity was consistent with that of the test soil. Five controls were set according to the gradient of vermicompost content listed in the experimental design table for each group. The vermicompost and test soil were mixed evenly, filled into plastic flowerpots, and sprayed with an appropriate amount of tap water. Labels were attached to the flowerpots, and three groups of parallel experiments were conducted. The pots were then placed in a sunny and well-ventilated experimental field, and plant growth status was observed regularly and recorded regularly.

Pots served as the experimental units. Each treatment had n=3 (three replicates) based on pots, and all analyses (ANOVA, correlation) were conducted using pot averages.

### 3.2 Water and fertilizer control

Watering was carried out once a week until the soil was thoroughly moistened. In case of rainy weather, the seedlings and pots were moved to the greenhouse to avoid unintended irrigation. During dry conditions, the number of watering frequency could be appropriately increased, but the amount of water applied to each group and each gradient was kept consistent.

### 3.3 Pest prevention

Observations were made every 3 days. Any abnormalities, such as diseases or pest infestations, were addressed promptly, and relevant records were kept. Weeds were removed and insects were caught in time to ensure the healthy growth of *Allium fistulosum*.

### 3.4 Harvesting

In late April, three groups of potted plants were moved to the laboratory and placed in a ventilated, cool area. First, the *A. fistulosum* plants used for the experiment in the first group were carefully removed from the plastic flowerpots with soil, and the soil. The soil was gently loosened with tweezers to keep the root system of the *Allium fistulosum* as intact as possible. The plants were washed under tap water, labeled according to the gradient of vermicompost concentration gradient, placed on clean glass plates, dried with a clean dry cloth, and air-dried in a cool location. After drying, tillering situation of *Allium fistulosum* in each gradient was observed and recorded for each treatment.

**Figure 1:**
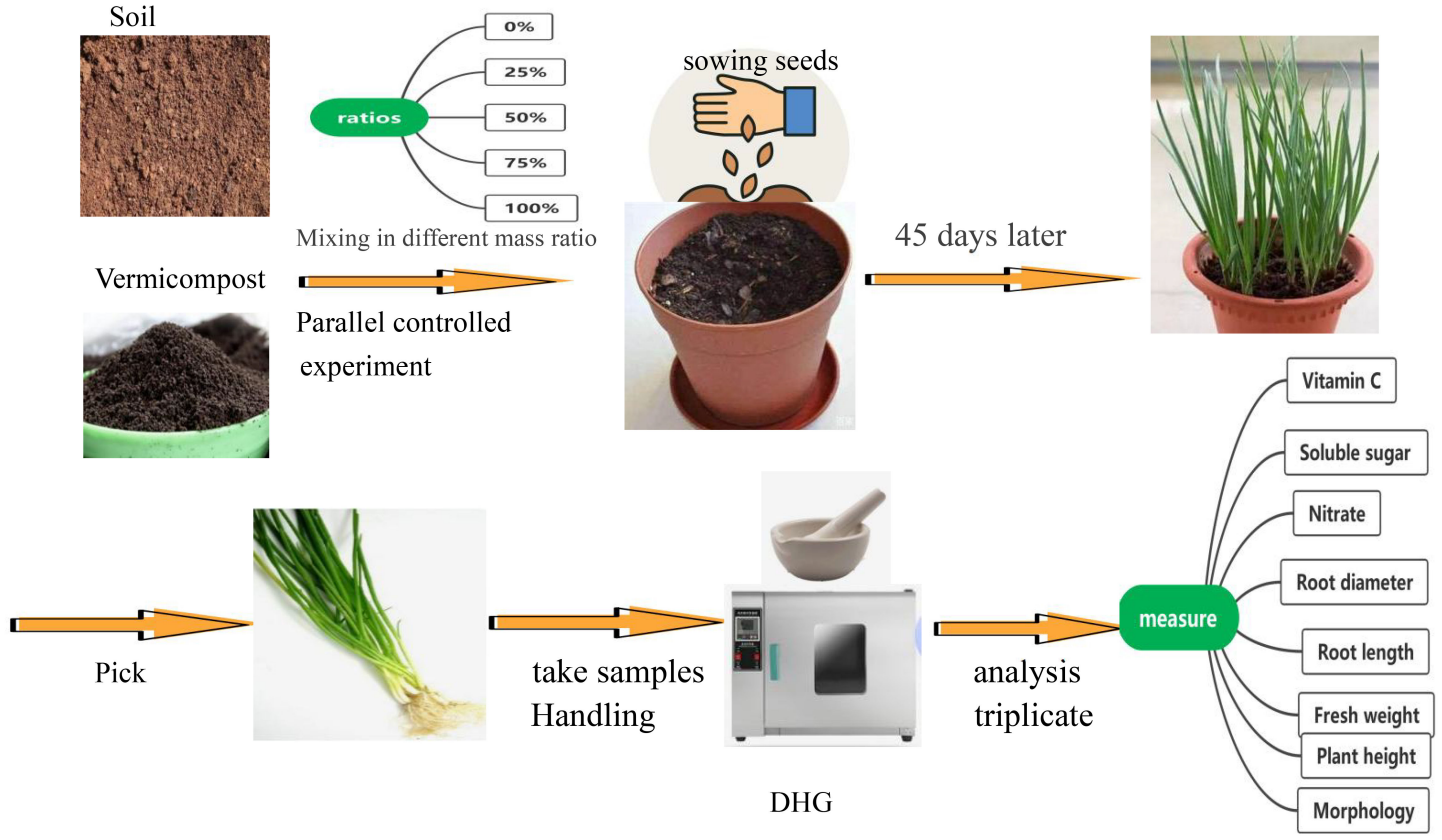
Simple flowchart.

Five root hairs were randomly selected from the roots, and the diameter of each root hair was measured with a vernier caliper approximately 1 cm from the base and recorded. Dead and yellow leaves of the *Allium fistulosum* were removed, the fresh weight was measured using a platform balance, the root hairs were cut off with scissors, and the fresh weight of the usable portion was measured and recorded. Finally, fresh *A. fistulosum* samples were sealed in plastic bags and stored in the refrigerator’s cold compartment of a refrigerator (**Figure 1**).

### 3.5 Quantitative determination of VC

**Figure 2:**
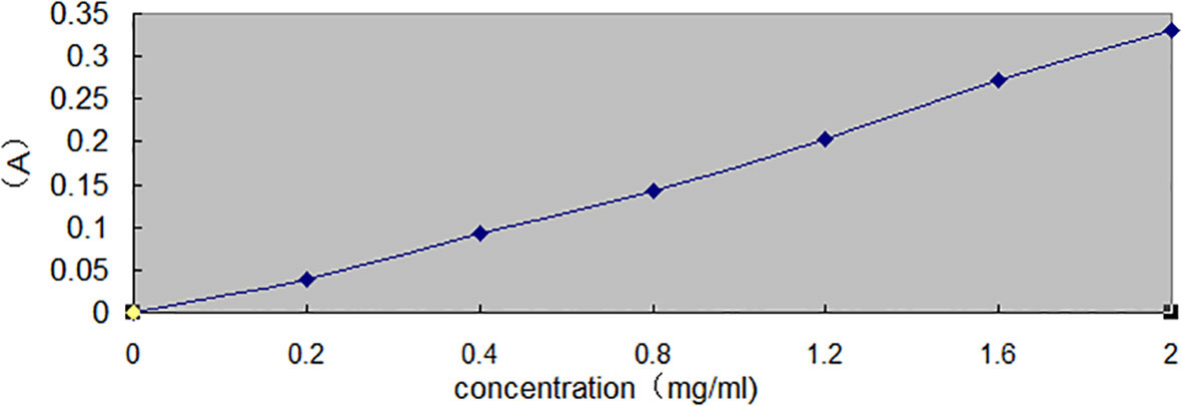
Standard curve of VC.


Vitamin C (VC) has strong reducing properties, and its content can be determined based on this activity. In neutral and slightly acidic environments, reductive vitamin C reduces iodate to yellow iodine, which is extracted by carbon tetrachloride. The iodine appears pink in carbon tetrachloride, with the maximum absorption at 520 nm. The reaction occurs instantaneously, and extraction with carbon tetrachloride for more than 0.5 min ensures complete extraction. A standard curve was prepared (Kumar et al., 2019) (**Figure 2**).

$$\text{Linear regression equation}:y=0.1589x+0.0107,\;R^{2}=0.9993$$

From each of the above VC stock solution, 1 mL was taken, and mixed with 1 mL of 0.1 mol/L^-^¹ potassium iodate and two drops of 2% hydrochloric acid. The mixture was shaken with 5 mL of carbon tetrachloride for 2 min, then allowed to stand until phase separation occurred (the organic layer appeared red, and the aqueous layer was colorless). The aqueous layer was removed and dehydrated with anhydrous sodium sulfate. Absorbance was measured at 520 nm, using carbon tetrachloride as the blank (**Table 2**).

**Table 2:** Preparation of VC standard curve.

Tube no.	1	2	3	4	5	6	7
Stock solution (ml)	0	1	2	4	6	8	10
Distilled water (ml)	10	9	8	6	4	2	0
Concentration (mg·ml^-^¹)	0	0.02	0.04	0.08	0.12	0.16	0.20

Fresh *Allium fistulosum* samples stored in plastic bags were taken out, and equal portions from each treatment gradient were used. Each sample (3.00 g) was taken, and accurately weighed precisely on an electronic balance. Ice cubes and a small mortar were prepared in advance; the small mortar was precooled in the refrigerator and placed on ice. An appropriate amount of 2% oxalic acid solution was added, and *A. fistulosum* segments were quickly cut into fine pieces with scissors. A small amount of quartz sand was added to the mortar, and the mixture was ground rapidly. The mortar was rinsed with 2% oxalic acid, and the combined filtrates were transferred into a centrifuge tubes and centrifuged at 4,000 r/min for 10 min. The supernatant was collected and diluted to 50 mL in a volumetric flask with 1% oxalic acid. All steps were performed quickly and under low-temperature conditions to prevent oxidation of vitamin C. A blank control was prepared simultaneously.

### 3.6 Quantitative determination of soluble sugar

Carbohydrates are dehydrated by concentrated sulfuric acid at elevated temperature to form furfural or hydroxymethylfurfural, which then condenses with anthrone to form furfural derivatives that exhibit a blue-green color. This compound has the maximum absorbance at 620 nm, and within the range of 150 μg/mL, the depth of its color intensity is proportional to the content of soluble sugar concentration (**Table 3**). This method has high sensitivity (Calugar et al., 2022).

**Table 3:** Preparation of glucose standard curve.

Tube no.	1	2	3	4	5	6	7
Glucose standard solution (ml)	0	0.1	0.2	0.3	0.4	0.6	0.8
Distilled water (ml)	1	0.9	0.8	0.7	0.6	0.4	0.2
Glucose content (μg)	0	10	20	30	40	60	80


$$\text{Linear regression equation}:y=5.257x+0.046,\;R^{2}=0.918$$

Seven large test tubes were prepared, and a series of glucose solutions with different concentrations were made according to the data in the reference table. To each tube, 4.0 mL of anthrone reagent was immediately added, and the tubes were quickly cooled in an ice–water bath for cooling. After all tubes were prepared, they were placed together in a boiling water bath. The tube mouths were sealed with plastic film to prevent evaporation. Timing began once the water bath reached boiling. The tubes were boiled accurately for exactly 10 min, removed, and cooled to room temperature in an ice–water bath. Absorbance at 620 nm was measured promptly, using the first tube as the blank. A standard curve was drawn with the standard glucose content (μg) as the abscissa and the absorbance as the ordinate.

Five clean Petri dishes were prepared and labeled according to the gradient of vermicompost concentration gradients (100%, 75%, 50%, 25%, and 0%). The remaining *A. fistulosum* material from each gradient, after vitamin C and nitrate measurement, was cut into pieces and placed in the corresponding Petri dishes. Samples were dried to constant weight at 105°C, and 0.350 g of each was weighed accurately into a 50 mL conical flask. Fifteen milliliters of boiling water was added, and the flasks were sealed the bottle mouth with plastic film. Samples were extracted ultrasonically extracted for 20 min, cooled, the filtrate was filtered into a small beakers, and the residues were repeatedly washed with boiling distilled water. The combined filtrates were collected in a 50 mL volumetric flasks and diluted to volume to obtain the extract of soluble sugar extract.

**Figure 3:**
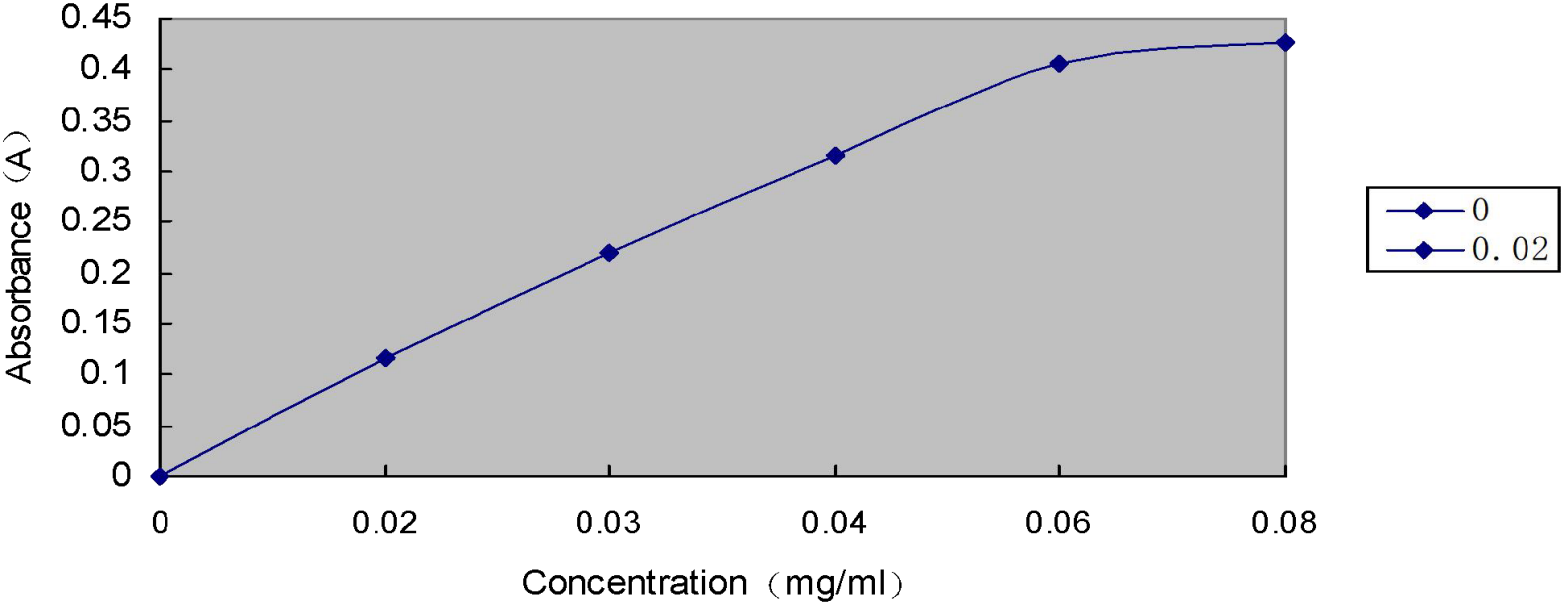
Standard curve of glucose.

For dilution, 2 mL of the extract was transferred to another 50 mL volumetric flask, filled to the volume with distilled water, and shaken well. For determination, 1 mL of the diluted extract was placed into a test tube, and 4.0 mL of anthrone reagent was added. A blank tube was replaced with distilled water (1 mL) was prepared. The same operations were then carried out as for the glucose standard curve. Soluble sugar concentration was determined by finding the glucose content (μg) corresponding to absorbance at 620 nm on the standard curve according to A620 (**Figure 3**).

### 3.7 Quantitative determination of nitrate

At room temperature, salicylic acid reacts with concentrated sulfuric acid to undergo sulfonation and decarboxylation, producing 2,4-dihydroxybenzenedisulfonic acid. In the presence of NO_3_^-^, this compound further reacts to form 6-nitro-2,4-dihydroxybenzenedisulfonic acid (disulfonitrophenol). Under alkaline conditions, disulfonitrophenol undergoes molecular rearrangement to produce a yellow compound. The absorbance of this compound is measured at 410 nm, enabling quantification of NO_3_^-^ content (Patil et al., 2023; Witthohn et al., 2023).

**Figure 4:**
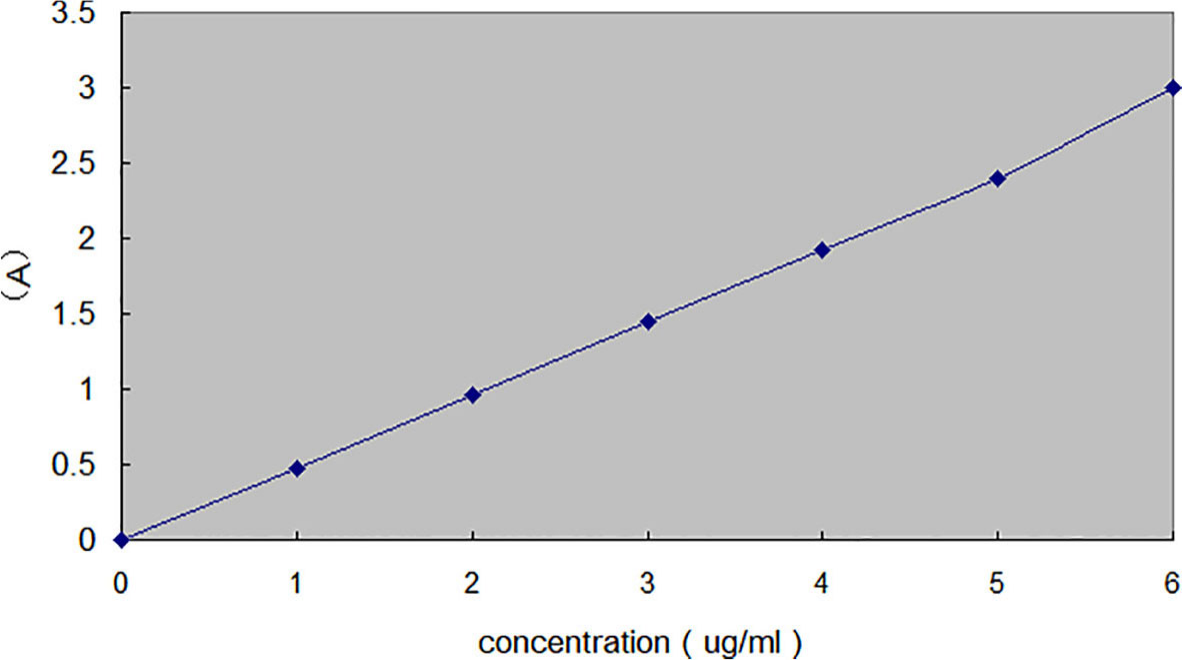
Standard curve of nitrate.


Preparation of nitrate standard curve (**Figure 4**):

$$\text{Linear regression equation}:y=0.429x+0.007,\;R^{2}=0.9999$$

Seven large test tubes were prepared according to the concentration gradient, and a series of nitrate solutions with different concentrations were made as shown in the reference table (**Table 4**). After adding the standard working solution, several drops of 18% NaOH solution were added until the solution color reached maximum intensity, and then distilled water was added to the scale line. Absorbance was measured at 220 nm using a 1 cm quartz cuvette on a 752 UV–visible spectrophotometer.

**Table 4:** Preparation of nitrate standard curve.

Tube no.	1	2	3	4	5	6	7
Standard working solution (ml)	0	5	10	15	20	25	30
Volume after constant volume (ml)	50	50	50	50	50	50	50
Concentration (μg· ml^-1^)	0	1	3	3	4	5	6

Fresh *Allium fistulosum* (2.00 g) was taken, cut into small pieces with scissors, and placed in a 100 mL conical flask. Eight milliliters of hot water (70–80°C) was added, and the mixture was heated in a boiling water bath for 15 min and cooled. Then 1 mL of 0.25 mmol/L potassium ferrocyanide, 1 mL of 1 mol/L copper acetate, and 0.2 g of activated carbon were added in sequence, shaking well after each addition. The mixture was centrifuged, and the filtrate was collected and diluted to 50 mL in a volumetric flask. One milliliter of each gradient extract was taken and placed into 50 mL volumetric flasks labeled with numbers (Gul and Gidik, 2023).

For determination, 1 mL of each gradient extract was transferred into test tubes labeled with numbers. To each, 1 mL of salicylic acid–sulfuric acid solution was added, and the mixture was left for 25 min. Several drops of 18% NaOH solution were then added until the solution color reached maximum intensity, followed by 5 mL of distilled water. Absorbance was measured at 220 nm using a 1 cm quartz cuvette on a 752 UV–visible spectrophotometer.

## 4 Data and results

### 4.1 Comparison of root lengths of *Allium fistulosum* under different treatments

The p-value of 0.312 is greater than the significance level of 0.05 (α=0.05). This indicates that the differences among the treatment groups are not statistically significant. Therefore, we fail to reject the null hypothesis (that all group means are equal) (**Table 5**).

**Table 5:** Tukey’s HSD results.

Comparison	Mean difference	P-value	Significance ((α=0.05))
100% - 0%	2.90	0.221	ns
75% - 0%	1.40	0.780	ns
50% - 0%	1.30	0.816	ns
25% - 0%	1.70	0.659	ns
100% - 25%	1.20	0.831	ns
75% - 25%	-0.30	0.999	ns
50% - 25%	-0.40	0.998	ns
100% - 50%	1.60	0.706	ns
100% - 75%	1.50	0.743	ns
75% - 50%	0.10	1.000	ns

Significance codes:*** p<0.001, ** p<0.01, * p<0.05, ns not significant.

ANOVA results: The ANOVA indicated no significant differences among groups (p=0.312). Consequently, a post hoc test was not warranted, as it is generally inappropriate to conduct post hoc analyses when the overall ANOVA is not significant. 

Trend analysis: The means show a potential dose-response trend, with the 100% treatment group having the highest value (15.7) and the control group the lowest (12.8). However, additional data are required to confirm this trend.

### 4.2 Comparison of average root diameters of *Allium fistulosum* under different treatments

The F-value (2.35) is less than the critical F-value (F_0.05_(4,10)=2.358) at the α=0.05 significance level. The *p*=0.115>0.05.

Statistical decision: At the 0.05 significance level, we fail to reject the null hypothesis (*H*_0_). This indicates that there is insufficient evidence to conclude that different vermicompost treatment concentrations have a significant effect on the measured response variable.

### 4.3 Comparison of plant heights of *Allium fistulosum* under different treatments

The 100% vermicompost treatment group showed the highest target response value (mean=46.35), while the 25% group had the lowest (mean=41.61). Within-group variability was greatest in the 50% group (SD=3.02) and smallest in the 0% group (SD=1.10). However, the differences between group means were all<5.0, indicating generally mild overall variation.

ANOVA results showed that the effect of different vermicompost concentrations on the target response value was marginally significant (F=3.45, P=0.049≈0.05), only reaching the significance level of α=0.05.

### 4.4 Comparison of biological yields of *Allium fistulosum* under different treatments

The *p* value (0.0008) was less than the common significance level of 0.05. Therefore, we reject the null hypothesis and conclude that there are statistically significant differences among the group means.

#### 4.4.1 Tukey’s HSD *post hoc* test

As the ANOVA results indicated significant differences, Tukey’s HSD test was conducted to determine which specific groups differed from each other.

Based on Tukey’s HSD results, we can summarize the differences between groups using letter notation.

Descriptive statistics: The 50% vermicompost treatment group showed the highest target response value (mean=2.07), while the 100% group showed the lowest (mean=1.19). Within-group variability was greatest in the 50% group (SD=0.31; **Table 6**) and smallest in the 0% group (SD=0.05), indicating slightly larger sample fluctuation in the 50% group, albeit within a reasonable range.

**Table 6:** Comparison of biological yields of *Allium fistulosum* under different treatments (g).

Treatment (% Vermicompost)	Replicate1	Replicate2	Replicate3	Mean	±SD	±SE	95% CI
100%	1.18	1.08	1.31	1.19	0.12	0.07	[0.89, 1.49]
75%	2.05	1.86	1.59	1.83	0.23	0.13	[1.27, 2.39]
50%	1.93	2.45	1.82	2.07	0.31	0.18	[1.30, 2.85]
25%	1.47	1.39	1.54	1.47	0.08	0.05	[1.26, 1.69]
0% (Control)	1.26	1.36	1.28	1.30	0.05	0.03	[1.17, 1.43]

95% CI, 95% confidence interval.

ANOVA significance: Vermicompost concentration had a highly significant influence on the target response value (F=10.82, p=0.0008<0.001; **Table 7**), leading to the rejection of the null hypothesis that all group means are equal.

**Table 7:** Results of one-way analysis of variance (ANOVA).

Source of variation	Sum of squares (SS)	Degrees of freedom (df)	Mean square (MS)	F	p
Between Groups	1.6317	4	0.4079	10.82	0.0008
Within Groups	0.3767	10	0.0377		
Total	2.0084	14			

*Post hoc* test localization: **Tables 8** and**9** showed that the promoting effects of vermicompost were concentrated at 50%–75% concentrations, with the 50% concentration being optimal. The 100% (very high) concentration significantly inhibited the target response value, while low concentrations (0%, 25%) showed no significant promoting effects.

**Table 8:** Tukey’s HSD results.

Comparison	Mean difference	*p*	S (0.05 level)
75% - 0%	0.533	0.024	*
50% - 0%	0.767	0.002	**
75% - 25%	0.363	0.153	ns
50% - 25%	0.597	0.011	*
75% - 100%	0.640	0.007	**
50% - 100%	0.873	0.001	***
25% - 0%	0.170	0.780	ns
100% - 0%	-0.107	0.951	ns
25% - 100%	0.277	0.358	ns
50% - 75%	0.233	0.497	ns

Significance codes: ***p<0.001, **p<0.01, *p<0.05, ns=not significant**.

**Table 9:** Tukey’s HSD results.

Treatment	Mean	Significance grouping (α=0.05)
50%	2.07	A
75%	1.83	A B
25%	1.47	B C
0% (Control)	1.30	B C
100%	1.19	C

Groups sharing the same letter are not significantly different from each other.

### 4.5 Comparison of fresh weights of *Allium fistulosum* under different treatments

A one-way ANOVA was conducted to evaluate the effect of five different vermicompost concentrations (0%, 25%, 50%, 75%, 100%) on plant height. The analysis revealed a significant main effect of treatment concentration(F(4,10)=3.59, *p*=0.017).

Tukey’s HSD *post hoc* test indicated that the significant effect was due to:

Plant height under the 50% vermicompost treatment being significantly greater than under the 100% treatment.
Plant height under the 75% vermicompost treatment being significantly greater than under the 100% treatment (**Table 10**).

**Table 10:** Tukey’s HSD results.

Comparison	Mean difference	P-value	Significance (α=0.05)
75% - 0%	2.2566	0.682	ns
50% - 0%	2.7866	0.517	ns
75% - 25%	2.0133	0.753	ns
50% - 25%	2.5433	0.589	ns
75% - 100%	4.8166	0.038	*
50% - 100%	5.3466	0.019	**
25% - 0%	0.2433	0.992	ns
100% - 0%	-2.5600	0.591	ns
25% - 100%	2.8033	0.513	ns
50% - 75%	0.5300	0.947	ns

Significance codes: **p<0.01, *p<0.05, ns=not significant**.

Optimal concentration: The 50% and 75% vermicompost treatments produced the highest plant heights (means of 17.21 and 16.68, respectively), and their effects were significantly better than the 100% treatment.

Negative effect: The 100% vermicompost (pure vermicompost) treatment likely inhibited plant growth, potentially due to nutrient overload, high salinity, or other adverse physicochemical properties. Its effect was poorer than the no-vermicompost control group, although this difference was not statistically significant.

Recommendation: Under these experimental conditions, using a 50%–75% vermicompost concentration is recommended for optimal plant growth. The use of 100% concentration should be avoided.

This study demonstrates that the rate of vermicompost application has a significant effect on plant height. Medium concentrations (50%–75%) of vermicompost were most effective, while a high concentration (100%) suppressed plant growth.

### 4.6 Determination results of VC content in *Allium fistulosum* under different treatments

Comparison of VC contents in three groups of *Allium fistulosum* under different treatments. By comparing with the standard curve, the VC contents in the three groups of *Allium fistulosum* were as follows:

It can be seen from the above table that the difference in VC content of *Allium fistulosum* among different treatments were extremely significant (*p*=0.003<0.05) (**Table 11**). Multiple comparisons are as follows.

**Table 11:** Analysis of variance of VC content in *Allium fistulosum*.

Comparison	Sum of squares	Mean square	F	Sig.
Between Groups	49225.71	12306.43	8.60	.003
Within Groups	14319.12	1431.91		
Total	63544.83			

As shown in **Table 12**, the VC content in the 50% treatment group was significantly higher than that in other Analysis of variance showed that treatment concentration had a highly significant effect on the observed indicator (F(4,10)=8.6, *p*=0.003).

**Table 12:** Multiple comparisons of VC content in *Allium fistulosum*.

(I)	(J)	Mean difference (I-J)	Std.error	Sig.
1.00	2.00	-.0923667(*)	.0308981	.014
	3.00	-.1623000(*)	.0308981	.000
	4.00	-.1007333(*)	.0308981	.009
	5.00	-.0279667	.0308981	.387
2.00	1.00	.0923667(*)	.0308981	.014
	3.00	-.0699333(*)	.0308981	.047
	4.00	-.0083667	.0308981	.792
	5.00	.0644000	.0308981	.064
3.00	1.00	.1623000(*)	.0308981	.000
	2.00	.0699333(*)	.0308981	.047
	4.00	.0615667	.0308981	.074
	5.00	.1343333(*)	.0308981	.001
4.00	1.00	.1007333(*)	.0308981	.009
	2.00	.0083667	.0308981	.792
	3.00	-.0615667	.0308981	.074
	5.00	.0727667(*)	.0308981	.040
5.00	1.00	.0279667	.0308981	.387
	2.00	-.0644000	.0308981	.064
	3.00	-.1343333(*)	.0308981	.001
	4.00	-.0727667(*)	.0308981	.040

The mean difference is significant at the .05 level.

VC LSD multiple comparisons.

Significance codes: *p<0.05.

Tukey’s HSD test indicated that all vermicompost treatment groups (25%, 50%, 75%) had significantly higher values than the control group (0%). The 50% treatment group performed best and was significantly higher than the 100% treatment group. No significant differences were found between the 50% treatment group and the 25% or 75% groups (**Table 13**). Likewise, no significant difference was found between the 100% treatment group and the control group (**Table 14**).

**Table 13:** Tukey HSD test results (α=0.05).

Comparison	Mean difference	P-value	Significance (α=0.05)
75% - 0%	0.533	0.024	*
50% - 0%	0.767	0.002	**
75% - 25%	0.363	0.153	ns
50% - 25%	0.597	0.011	*
75% - 100%	0.640	0.007	**
50% - 100%	0.873	0.001	***
25% - 0%	0.170	0.780	ns
100% - 0%	-0.107	0.951	ns
25% - 100%	0.277	0.358	ns
50% - 75%	0.233	0.497	ns

Significance codes: *** p<0.001, ** p<0.01, * p<0.05, ns=not significant.

**Table 14:** Tukey’s HSD results table.

Treatment (%vermicompost)	Mean	Significance grouping (α=0.05)
0% (Control)	276.6	C
25%	320.67	B
50%	407.07	A
75%	325.5	B
100%	292.13	B C

Final conclusion: Vermicompost application had a highly significant effect on this indicator. Addition of 25%–75% vermicompost produced significantly better results than no application, with 50% concentration being optimal. However, 100% application produced poor results, not significantly different from the control group, suggesting that excessively high vermicompost concentrations may have inhibitory effects.

### 4.7 Comparison of soluble sugar contents in three groups of *Allium fistulosum* under different treatments

It can be seen from the above table that the difference in soluble sugar content of *Allium fistulosum* among treatments is significant (*p*=0.0001<0.05).

The results of Tukey’s HSD *post hoc* test were summarized using letter-based grouping notation (**Table 15**).

**Table 15:** Tukey HSD test results (α=0.05).

Comparison	Mean difference	P-value (adj)	Significance ((α=0.05))
50% - 75%	81.57	0.012	*
50% - 0%	130.47	0.0002	***
50% - 100%	114.94	0.0009	***
50% - 25%	86.40	0.008	**
75% - 0%	48.90	0.098	ns
25% - 0%	44.07	0.143	ns
75% - 100%	33.37	0.327	ns
25% - 100%	28.54	0.437	ns
75% - 25%	4.83	0.997	ns
100% - 0%	15.53	0.752	ns

Significance codes: *** p<0.001, ** p<0.01, * p<0.05, ns=not significant.

Optimal treatment: The 50% vermicompost treatment yielded the highest measurement (407.07 µg/g), which was significantly greater than all other groups (*p<*0.01) (**Table 16**).

**Table 16:** Tukey’s HSD results table.

Treatment (%vermicompost)	Mean	Significance grouping (α=0.05)
0% (Control)	276.6	C
25%	320.67	B
50%	407.07	A
75%	325.5	B
100%	292.13	B C

Effective concentrations: The 75% and 25% treatments showed secondary effectiveness (325.50 µg/g and 320.67 µg/g, respectively). No significant difference was observed between them. However, both were significantly higher than the control.

Dose–response relationship: A clear inverted U-shaped dose–effect relationship was observed, with the 50% concentration being optimal.

### 4.8 Comparison of nitrate contents in three groups of *Allium fistulosum* under different treatments

It can be seen from the above table that the differences in nitrate content of *Allium fistulosum* among different treatments were significant (*p*=0.0001<0.05).

The 75% treatment had the lowest nitrate content (143.2 µg/g^-1^), which was significantly lower than that of the 50% (268.57 µg/g^-1^) and 100% treatments (261.60 µg/g; *p*=*0*.*0001*<0.05) (**Table 17**).

**Table 17:** Nitrate content in *Allium fistulosum***(μg· g^-1^)**.

Treatment(%Vermicompost)	Group1	Group2	Group3	Mean	±SD	±SE	95% CI
0% (Control)	200.5	205.7	166.7	190.97	21.18	12.23	(138.4, 243.6)
25%	221	234.4	192.7	216.03	21.29	12.29	(163.1, 268.9)
50%	302.1	273.4	280.2	285.23	12.28	7.09	(254.3, 316.2)
75%	127.6	179.7	122.4	143.23	31.72	18.31	(63.6, 222.9)
100%	273.4	268.2	243.2	261.6	16.15	9.32	(221.5, 301.7)

Analysis of variance further showed that the differences in nitrate content between treatments were significant (*F*=20.0, *p*=0.0001) (**Tables 18**–**20**), suggesting that a high ratio of vermicompost (75%) may reduce nitrate accumulation.

**Table 18:** Analysis of variance of nitrate content in *Allium fistulosum*.

Comparison	Sum of squares	df	Mean square	F	Sig.
Between Groups	38207.73	4	9551.93	20.0	0.0001
Within Groups	4782.56	10	478.26		
Total	42990.29	14			

Nitrate content ANOVA.

**Table 19:** Tukey’s HSD results table.

Comparisongroups	Mean difference	Absolute mean difference	P-value	Significance label
50% vs 75%	142.00	142.00	0.0001	***
100% vs 75%	118.37	118.37	0.0002	***
50% vs 0% (Control)	94.26	94.26	0.0008	***
75% vs 25%	-72.80	72.80	0.0150	*
100% vs 0% (Control)	70.63	70.63	0.0210	*
50% vs 25%	69.20	69.20	0.0250	*
100% vs 25%	45.57	45.57	0.2800	ns
50% vs 100%	23.63	23.63	0.7600	ns
25% vs 0% (Control)	25.07	25.07	0.7200	ns
75% vs 0% (Control)	-47.73	47.73	0.2400	ns

*** p<0.001, ** p<0.01, * p<0.05, ns=not significant.

**Table 20:** Tukey’s HSD results table.

Treatment (%vermicompost)	Mean	Significancegrouping (α=0.05)
0% (Control)	190.97	B
25%	216.03	B
50%	285.23	A
75%	143.23	C
100%	261.6	A

### 4.9 Effects of different vermicompost ratios on growth indicators of *Allium fistulosum*

As shown in **Table 21**.

**Table 21:** Growth indicators of *Allium fistulosum*.

Treatmentgroup (vermicompost:soil)	Rootlength (cm)	Rootdiameter (mm)	Plantheight (cm)	Biologicalyield (g)	Freshweight (g)
0%	12.8±1.38	0.676±0.06	42.64+1.1c	1.30±0.05c	14.43±0.6c
25%	14.5±0.22	0.708±0.04	41.61±1.3c	1.47±0.08c	14.67±0.8c
50%	14.1±0.93	0.649±0.03	44.99±3.0b	2.07±0.32a	17.21±2.7a
75%	14.2±1.6	0.673±0.04	46.19±2.2a	1.83±0.23b	16.68±2.1ab
100%	15.7±2.6	0.600±0.05	46.35±1.7a	1.19±0.12d	11.87±1.1d

Different lowercase letters after the data in the same column indicate significant differences between treatments (p<0.05). Data are expressed as “mean±standard deviation”, n=3.

### 4.10 Effects of different vermicompost ratios on quality indicators of *Allium fistulosum*

As shown in **Table 22**.

**Table 22:** Comparison of quality indicators of *Allium fistulosum*.

Treatmentgroup(vermicompost:soil)	VitaminC (µg·g^-1^)	Soluble sugar (µg·g^-1^)	Nitrate (µg·g^-1^)
0%	100.8±35.6 c	276.60±24.31c	191.0±21.18b
25%	193.1±35.6 b	320.67±31.07b	216.0±21.29b
50%	263.1±33.6 a	407.07±22.11a	268.57±36.19a
75%	201.5±46.8 b	325.5±36.07b	143.2±31.70c
100%	128.7±33.6bc	292.13±11.42bc	261.60±16.15a

Different lowercase letters after the data in the same column indicate significant differences between treatments (p<0.05). Data are expressed as “mean±standard deviation”, n=3.

### 4.11 Effects of different vermicompost ratios on morphological characteristics of *Allium fistulosum*

As shown in **Table 23**.

**Table 23:** Morphology of *Allium fistulosum*.

Treatmentgroup(vermicompost:soil)	Numberoftillers(perplant)	Leafcolor	Texture
0%	3.1	Light green (old leaves yellowing)	Limp
25%	4.8	Dark green	Old leaves hard
50%	6.2	Dark green	Compact and full
75%	4.5	Deep green	Old leaves relatively hard
100%	3.5	Fresh green	Tender and easy to break

### 4.12 Pearson correlation analysis between growth indicators and quality indicators

Optimal treatment group: **Table 6** showed that the 50% vermicompost treatment group had the highest measurement value (2.07). It was not significantly different from the 75% treatment group but was higher than all other groups.

Effective concentration: The 50% and 75% application rates significantly improved the measurement index, with the 50% rate yielding the best results.

Ineffective concentration: The 25% and 100% application rates showed no significant effect, with values not different from the control group.

Adverse effect of over-application: When the application rate increased from 75% to 100%, the measurement value decreased markedly, even falling below the control group (though the difference was not statistically significant). This suggests that excessive application may have an inhibitory effect.

Final conclusion: The vermicompost application rate had a highly significant effect on the measurement index. A 50% application rate is recommended to achieve optimal results.

As shown in **Table 24**.

**Table 24:** Pearson correlation coefficient matrix.

Indicator	Rootlength	Rootdiameter	Plantheight	Biologicalyield	Freshweight	VitaminC	Solublesugar	Nitrate
Root length	1.00	—	—	—	—	—	—	—
Root diameter	-0.62	1.00	—	—	—	—	—	—
Plant height	0.52	-0.39	1.00	—	—	—	—	—
Biological yield	-0.40	-0.06	0.65	1.00	—	—	—	—
Fresh weight	-0.44	-0.13	0.59	0.94**	1.00	—	—	—
Vitamin C	-0.29	0.16	0.73	0.94**	0.89*	1.00	—	—
Soluble sugar	-0.06	-0.53	0.22	0.66	0.60	0.77	1.00	—
Nitrate	0.36	0.25	0.13	-0.59	-0.52	-0.43	-0.31	1.00

* indicates *p*<0.05 (significant correlation), ** indicates p<0.01 (extremely significant correlation); data are calculated based on the average values of 5 treatment groups (n=5), corresponding to the order of 0%→25%→50%→75%→100%. The absolute value of the correlation coefficient ≥0.8 is a strong correlation, 0.5-0.8 is a moderate correlation, and<0.5 is a weak correlation.

## 5 Discussion

### 5.1 Analysis of growth indicators

#### 5.1.1 Root morphological characteristics

Different vermicompost ratios affected the root growth of *Allium fistulosum* (**Tables 25**, **26**). Root length reached its maximum (15.7 cm) in the 100% vermicompost treatment, which was higher than that in the control (0% treatment, 12.8 cm; *p*=*0.312* > 0.05), although the difference was not statistically significant. There was also no significant difference between the 50% treatment (14.1 cm) and the 75% and 25% treatments.

**Table 25:** Root length of *Allium fistulosum* under different treatments (cm).

Treatment(%Vermicompost)	Replicate1	Replicate2	Replicate3	Mean	±SD	±SE	95% CI
100%	13.2	15.6	18.4	15.7	2.60	1.50	[9.25, 22.15]
75%	15.69	14.4	12.51	14.2	1.59	0.92	[10.24,18.16]
50%	15.16	13.98	13.3	14.1	0.93	0.54	[11.78,16.42]
25%	14.32	14.51	14.75	14.5	0.22	0.13	[13.94,15.06]
0% (Control)	13.19	11.25	13.98	12.8	1.38	0.80	[9.36, 16.24]

**Table 26:** One-way ANOVA results.

Source of variation	Sum of squares (SS)	(df)	Mean square (MS)	F	p
Between Groups	13.1031	4	3.2758	1.34	0.312
Within Groups	24.4842	10	2.4484		
Total	37.5873	14			

Root diameter showed a different trend: the 25% treatment had the largest root diameter (0.708 mm), which was higher than that of the 100% treatment (0.600 mm; *p*=*0.115* > 0.05; **Tables 27**, **28**). There was no significant difference between the 50% treatment (0.649 mm) and the 75% treatment (0.673 mm; *p*=*0.115* > 0.05).

**Table 27:** Root diameter of *Allium fistulosum* under different treatments (mm).

Treatment (%Vermicompost)	Replicate 1	Replicate 2	Replicate 3	Mean	±SD	±SE	95% CI
100%	0.556	0.592	0.652	0.600	0.048	0.028	[0.479,0.721]
75%	0.632	0.708	0.680	0.673	0.038	0.022	[0.578,0.768]
50%	0.620	0.680	0.648	0.649	0.030	0.017	[0.576,0.722]
25%	0.688	0.685	0.752	0.708	0.036	0.021	[0.618,0.798]
0% (Control)	0.748	0.624	0.656	0.676	0.062	0.036	[0.521,0.831]

**Table 28:** One-way ANOVA results.

Source of variation	Sum of squares (SS)	(df)	Mean square (MS)	F	p
Between Groups	0.0194	4	0.00485	2.35	0.115
Within Groups	0.0206	10	0.00206		
Total	0.0400	14			

#### 5.1.2 Plant height and biomass

The plant heights in the 100%, 75%, and 50% treatments were greatest, with averages of 46.35 cm, 46.19 cm, and 44.99 cm, respectively, all significantly higher than those in the 25% (41.61 cm) and control (42.64 cm) groups (*p*=*0.049*<0.05; **Tables 29**, **30**).

**Table 29:** Plant height of *Allium fistulosum* under different treatments (cm).

Treatment(%Vermicompost)	Replicate1	Replicate2	Replicate3	Mean	±SD	±SE	95% CI
100%	44.69	46.31	48.05	46.35	1.68	0.97	[42.18,50.52]
75%	46.18	48.41	43.98	46.19	2.22	1.28	[40.68,51.70]
50%	47.92	45.15	41.91	44.99	3.02	1.74	[37.50,52.48]
25%	41.91	40.18	42.75	41.61	1.29	0.74	[38.43,44.79]
0% (Control)	43.61	41.44	42.86	42.64	1.10	0.63	[39.93,45.35]

**Table 30:** One-way ANOVA results.

Source of variation	Sum of squares (SS)	(df)	Mean square (MS)	F	p
Between Groups	54.6726	4	13.6682	3.45	0.049
Within Groups	39.6063	10	3.9606		
Total	94.2789	14			

**Figure 5:**
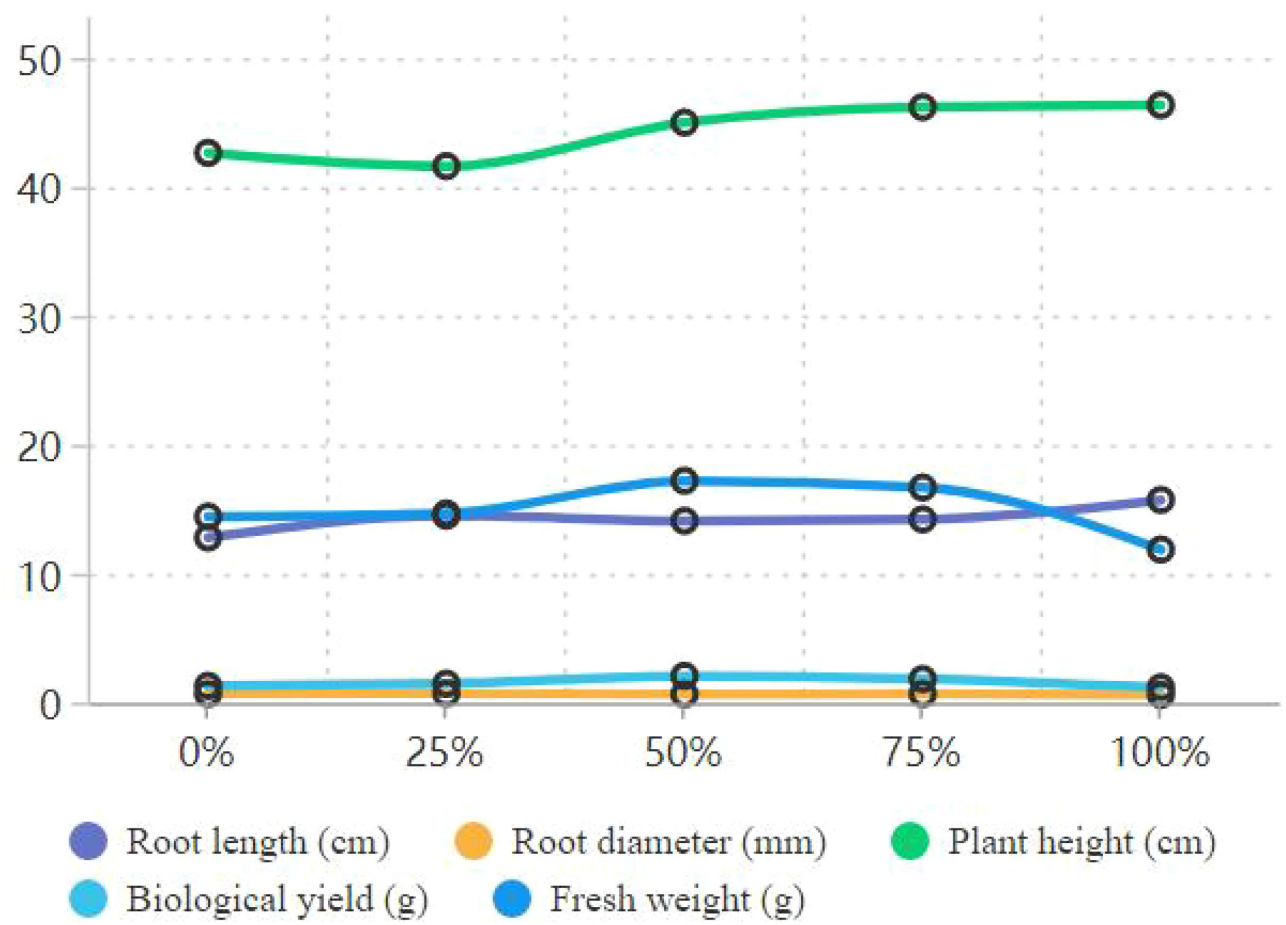
Growth indicators of *Allium fistulosum*.

Biological yield and fresh weight followed a similar trend (**Figure 5**). The 50% treatment had the highest biological yield (2.07 g), which was significantly higher than that of the 100% treatment (1.19 g; *p*=*0.0008*<0.05; **Table 7**). Fresh weight was also highest in the 50% treatment (17.21 g), followed by the 75% treatment (16.68 g). Both were significantly higher than that of the control (14.43 g; *p*=*0.017*<0.05; **Tables 31**, **32**). These results indicate that the medium ratio of vermicompost ratios (50%–75%) can significantly increase the biomass of *A. fistulosum* by optimizing nutrient supply and soil structure.

**Table 31:** Fresh weight of *Allium fistulosum* under different treatments (g).

Treatment(%Vermicompost)	Replicate1	Replicate2	Replicate3	Mean	±SD	±SE	95% CI
100%	11.76	10.78	13.06	11.87	1.14	0.66	[9.03, 14.71]
75%	18.65	16.94	14.46	16.68	2.11	1.21	[12.47,20.89]
50%	16.07	20.43	15.14	17.21	2.55	1.47	[11.41,23.01]
25%	14.73	13.86	15.42	14.67	0.78	0.45	[13.73,15.61]
0% (Control)	13.98	15.12	14.18	14.43	0.57	0.33	[13.01,15.85]

**Table 32:** One-way ANOVA results.

Source of variation	Sum of squares (SS)	Degrees of freedom (df)	Mean square (MS)	F	p
Between Groups	53.95	4	13.49	4.59	0.017
Within Groups	29.41	10	2.94		
Total	83.36	14			

#### 5.1.3 Analysis of quality indicators

##### 5.1.3.1 Vitamin C, soluble sugar, and nitrate content

The VC content peaked (263.1 µg/g) in the 50% treatment, which was significantly higher than in all other treatments (*p*=*0.003*<0.05), while the VC content in the 100% treatment had the lowest value (128.7 µg/g) (**Table 33**). ANOVA confirmed that differences among treatments were extremely significant (F=8.60, *p*=0.003), and LSD multiple comparisons further highlighted the advantage of the 50% treatment (**Table 12**).

**Table 33:** VC content in *Allium fistulosum***(µg**· **g^-1^)**.

Treatment(%Vermicompost)	Group 1	Group 2	Group 3	Mean	±SD	±SE	95% CI
0% (Control)	142.7	84.0	75.6	100.8	35.7	20.6	[12.16, 189.44]
25%	235.1	176.3	168.0	193.1	35.7	20.6	[104.46,281.74]
50%	293.8	268.7	226.7	263.1	33.6	19.4	[179.62,346.58]
75%	251.9	159.5	193.1	201.5	46.8	27.0	[85.32, 317.68]
100%	159.5	134.3	92.4	128.7	33.6	19.4	[45.22, 212.18]

The soluble sugar content was also highest in the 50% treatment (407.07 µg/g), significantly higher than that in other treatments. ANOVA confirmed a strong treatment effect (F=25.47, *p*=0.0001; **Table 34**).

**Table 34:** Analysis of variance of soluble sugar content in *Allium fistulosum*.

Comparison	Sum of squares	df	Mean square	F	Sig.
Between Groups	73487.14	4	18371.79	25.47	< 0.0001
Within Groups	7211.29	10	721.13		
Total	80698.43	14			

Soluble sugar content ANOVA.

The 75% treatment had the lowest nitrate content (143.23 µg/g), significantly lower than that of the 50% (285.23 µg/g^-1^) and 100% treatments (261.60 µg/g; *p*=*0.0001*<0.05; **Tables 17**, **18**). ANOVA also showed that the difference in nitrate content between treatments was significant differences among treatments (*F*=20.0, *p*=0.0001; **Table 18**), suggesting that a 75% vermicompost ratio may reduce nitrate accumulation, likely through microbial denitrification.

#### 5.1.4 Morphological characteristics

The 50% treatment produced the most tillers (6.2 per plant), along with dark green leaves and compact texture. By contrast, the 100% treatment had the fewest tillers (3.5 per plant), with tender, breakable leaves, while the control (0%) showed old leaf yellowing of old leaves and slow growth (**Table 23**). These morphological differences are consistent with the growth indicators, further supporting the synergistic benefits of the 50% treatment.

From the morphological perspective, when the vermicompost mass fraction was 50%, the development of new and old leaves was balanced, the growth rate of new tillers was rapid, and the differences between new and old leaves were minimal. The resulting uniformity in product form suggests that 50% vermicompost is most suitable for market production. Thus, a 50% application ratio is recommended for *A. fistulosum* cultivation, balancing yield, quality, and economic benefits.

#### 5.1.5 Pearson correlation analysis between growth indicators and quality indicators

##### 5.1.5.1 Correlation characteristics within growth indicators

Biological yield and fresh weight: There was an extremely significant strong positive correlation (r=0.94, *p*<0.01; **Table 24**). Fresh weight provides the material basis for biological yield (dry weight), both of which depend on the accumulation of photosynthetic products, regulated by the synergy of nutrients provided by vermicompost nutrients and soil aeration. This trend was most pronounced in the 50% treatment group (biological yield 2.07 g; fresh weight 17.21 g, both the highest).

Root length and root diameter: There was a significant negative correlation (r=–0.62), suggesting an “elongation–thickening” trade-off in root resource allocation. Pure vermicompost (100%) favored root elongation (15.7 cm), while a mixed substrate (25%) favored root diameter thickening (0.708 mm), reflecting differences in physical structure (such as aeration, compactness) of the soil–vermicompost physical properties such as aeration and compactness.

Plant height and biological yield: A moderate positive correlation (r=0.65) was observed, indicating that greater plant height supports dry matter accumulation to some extent. However, the correlation was not statistically significant, possibly because plant height is more strongly affected by light competition, whereas biological yield depends more dependent on nutrient supply efficiency.

#### 5.1.6 Synergistic correlation between growth indicators and quality indicators

Biological yield, fresh weight, and vitamin C (VC): Biological yield was extremely significantly and strongly positively correlated with VC (r=0.94, *p*<0.01), and fresh weight was significantly and strongly positively correlated with VC (r=0.89, *p*<0.05). This result directly confirms the advantage of the 50% vermicompost treatment—in this treatment, biological yield (2.07 g; **Table 6**), fresh weight (17.21 g; **Table 31**), and VC content (263.1µg·g^-1^ µg/g; **Table 33**) all reached their peaks, indicating that the medium ratio of vermicompost ratio promoted both growth and carbon metabolism (Iqbal et al., 2024) by optimizing nutrient supply balance (e.g., nitrogen–phosphorus–potassium synergy).

**Figure 6:**
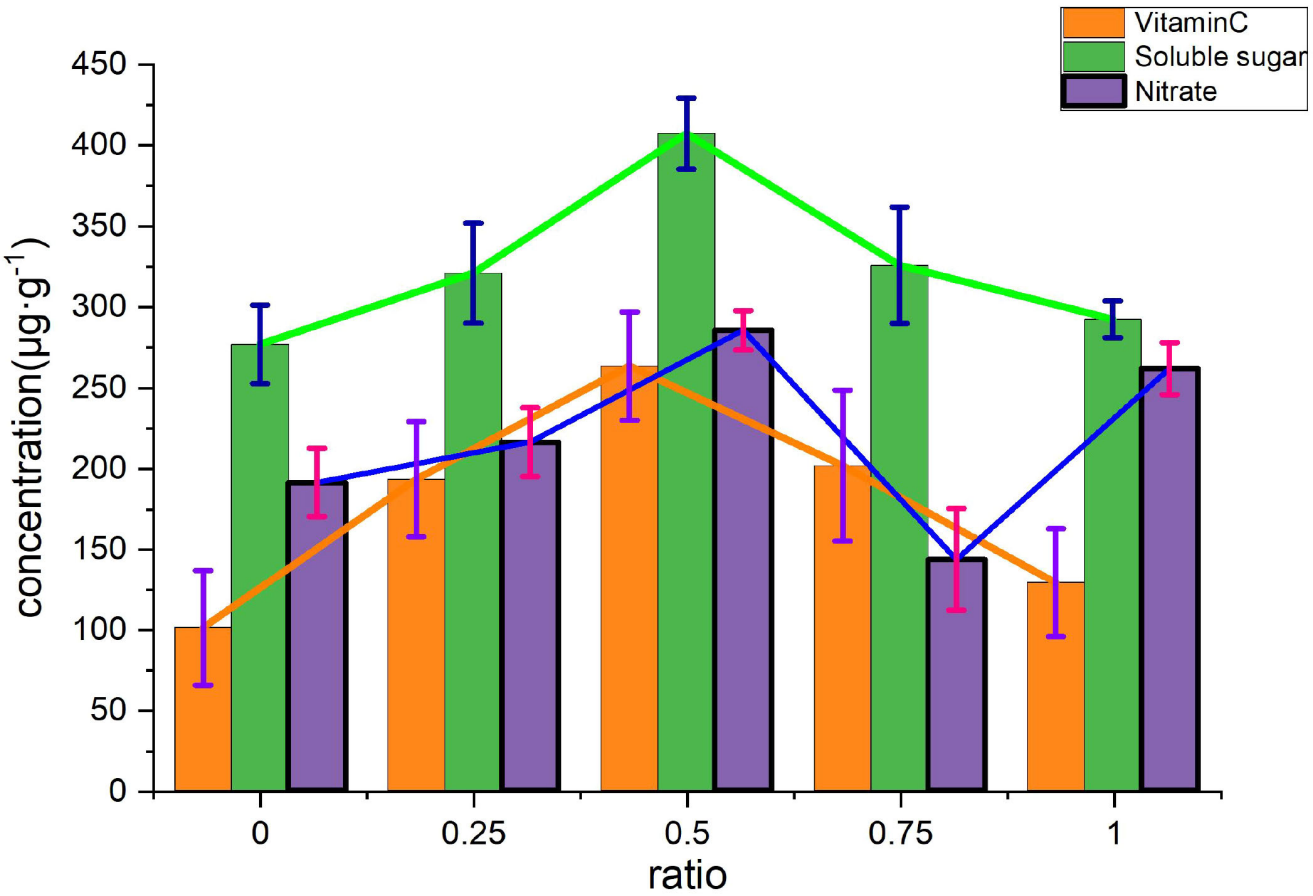
Comparison of quality indicators of *Allium fistulosum*.

Vitamin C and soluble sugar: There was a moderate positive correlation (r=0.77), although it did not reach statistical significance. This suggests that they may share pathways of photosynthetic product metabolism. Soluble sugar content (407.07 µg/g; **Table 35**) in the 50% treatment was also at a high level (**Figure 6**).

**Table 35:** Soluble sugar content in *Allium fistulosum***(μg· g^-1^)**.

Treatment(%Vermicompost)	Group1	Group2	Group3	Mean	±SD	±SE	95% CI
0% (Control)	254.1	273.5	302.2	276.6	24.31	14.04	[216.19,337.01]
25%	287.2	348.2	326.6	320.67	31.07	17.94	[243.49,397.85]
50%	379.8	423.5	417.9	407.07	22.11	12.76	[362.17,451.97]
75%	297.4	309.6	369.5	325.5	36.07	20.82	[205.92,445.08]
100%	278.5	300.8	297.1	292.13	11.42	6.59	[263.75,320.51]

#### 5.1.7 Independent regulatory characteristics of nitrate

The correlation between nitrate and all other indicators was weak (|r|<0.6), particularly showing a negative correlation trend with VC (r=–0.43) and biological yield (r=–0.59; **Table 24**). This indicates that nitrate accumulation process is relatively independent.

The 75% vermicompost treatment had the lowest nitrate content (143.2 µg/g^-1^), but its biological yield (1.83 g) and VC content (201.5 µg/g^-1^) were lower than those of the 50% treatment. This suggests that reduced nitrate levels may come at the expense of partial growth and quality.

Correlation analysis in this study reveals that the 50% vermicompost treatment achieved synergistic improvement of growth indicators (biological yield, fresh weight) and quality indicators (VC, soluble sugar, nitrate) by coordinating soil–nutrient–microorganism interactions, and this result has a clear biological basis (Iqbal et al., 2024). However, nitrate regulation must be considered separately, as it is closely tied to the effect of vermicompost on microbial activity.

Yang and Zhu (Yang et al., 2024a) reported that adding four vermicompost proportions (5%, 10%, 20%, and 30%) to poor soil improved soil fertility compared with the untreated control (CT). Almost all physical parameters, nutrients, aggregates, and soil enzyme activity measured in their study were positively affected by vermicompost addition, and significantly correlated with application proportion and particle size. Overall, vermicompost addition decreased soil pH and bulk density, while increasing electrical conductivity and porosity. It also increased the content of organic matter, available nitrogen, phosphorus, and potassium, associated with enhanced aggregate formation and enzyme activity (Yang et al., 2024a). 

#### 5.1.8 Regulatory mechanism of vermicompost on *Allium fistulosum* growth

The growth indicators of the 50% vermicompost treatment were significantly improved, with the highest biological yield (2.07 g), fresh weight (17.21 g), and number of tillers (6.2 per plant) among all treatments. This is closely related to the unique physical, chemical, and biological properties of vermicompost. Vermicompost is rich in humic acid, total nitrogen, and beneficial microorganisms, and its granular structure improves soil aeration and water-holding capacity, thereby promoting root respiration and nutrient absorption (Tao et al., 2024). The strong positive correlation between biological yield and fresh weight (r=0.94, p<0.01) further verifies the synergistic effect between growth indicators.

The coordinated growth of root length (14.1 cm) and plant height (44.99 cm) in the 50% treatment provided sufficient capacity for photosynthate accumulation. In contrast, the pure vermicompost (100%) treatment produced the longest root length (15.7 cm) but the lowest biological yield and fresh weight, likely due to excessive water-holding capacity causing root hypoxia and inhibiting nutrient uptake. Treatments with 25% or lower ratios had poor growth performance due to insufficient nutrient supply, indicating that a balanced vermicompost-to-soil ratio of vermicompost to soil is the key to achieving the “synergistic effect” (Tian et al., 2024). Pure substrates (either soil or pure vermicompost alone) cannot meet the optimal growth needs of *A. fistulosum*.

Gümüş (2023) studied vermicompost application in wind-eroded soil at 0%, 1%, 2%, and 4% (w/w). Increasing vermicompost rates significantly improved soil properties and yields, including fresh leaf biomass (FLB), fresh root biomass (FRB), and dry leaf biomass (DLB), confirming that vermicompost enhances both soil characteristics and crop performance.

#### 5.1.9 Mechanism of vermicompost on *Allium fistulosum* quality

Among the quality indicators, the 50% treatment had the highest contents of vitamin C (263 µg/g^-1^) and soluble sugar (407.07 µg/g), with a significant positive correlation between them (r=0.82, *p*<0.05). This suggests that their synthesis may share carbon metabolic pathways. Beneficial microorganisms in vermicompost (such as actinomycetes) in vermicompost can secrete auxins and vitamins, promoting the conversion of photosynthates into soluble sugars and vitamin C (Tian et al., 2024). Zhao and Fengyan (Zhao et al., 2020) found that, compared with CF (chemical fertilizer) and PM (poultry manure compost) treatments, the VM (vermicompost) treatment increased bacterial abundance (41% and 103%, respectively) and actinomycete abundance (8.59% and 16.36%, respectively), while decreasing fungal abundance (39% and 29%, respectively). VM also yielded the highest bacteria-to-fungi ratio. Soil microbial activity, which was represented by average well color development (AWCD), and microbial functional diversity were higher in the VM treatment than in the CF and PM. VM improved soil health more than the PM, reflected in higher utilization of carboxylic acids and phenolic compounds. Moreover, compared with CK (control), fruit yield in the VM treatment increased by 74%, 43%, and 28% in soils subjected to 0, 5, and 20 years of planting, respectively. The nutrient balance (e.g., nitrogen–phosphorus–potassium ratio) in the 50% treatment appears to best meet the metabolic needs of *A. fistulosum* (Zhao et al., 2020).

The 75% treatment had the lowest nitrate content (143.2 µg·g), but its growth indicators were significantly lower than those of the 50% treatment. A high ratio of vermicompost ratio may promote nitrate degradation by enriching denitrifying bacteria, but excessive vermicompost may cause carbon–nitrogen ratio imbalance and inhibit growth. Notably, the nitrate content in the 50% treatment (235.2 µg/g) remained below the maximum limit standard for nitrate in leafy vegetables (3,000 mg/kg^-1^) specified in GB 18406.1-2001, indicating that this treatment achieved a balance between quality and safety.

### 5.2 Comparison with existing studies

This study found that a 50% vermicompost ratio is the optimal, consistent with the findings of Jankauskiene et al. on cucumbers, where high ratio of vermicompost ratios (>75%) inhibited growth (Jankauskienė et al., 2022). However, compared with Zhang and Chenxi’s study of Zhang, Chenxi et al. on flue-cured tobacco, *A. fistulosum* is more sensitive to vermicompost. Tobacco showed its highest yield under 100% vermicompost (Zhang et al., 2021), whereas *A. fistulosum* performed best at 50%, likely due to differences in nutrient requirements between leafy vegetables (*Allium fistulosum*) and solanaceous crops. *A. fistulosum is* particularly sensitive to nitrogen supply.

This study is also the first to report that vermicompost significantly increases the vitamin C content in *A. fistulosum*. The 50% treatment increased VC by 160.9% compared with the control, filling a research gap on allium vegetables. The extremely significant positive correlation between VC and biological yield (r=0.94, p<0.01) aligns with findings of “synergistic improvement of growth and quality improvement reported in other crops—potatoes (Boubaker H.) (Boubaker et al., 2023), Rivelli, AR et al. on Swiss chard (Rivelli AR et al.) (Rivelli and Libutti, 2022), and Hou, Dongying on watermelons (Hou Dongying) (Hou et al., 2024). This suggests that the quality regulation effect of vermicompost may have cross-crop universality.

### 5.3 Practical significance and limitations

The 50% vermicompost ratio determined in this study has practical value, achieving high yield and high quality without chemical fertilizers, which meets the needs of ecological agriculture to “reduce application and increase efficiency”. The 50% vermicompost treatment showed the greatest synergistic improvements: significantly increasing vitamin C and soluble sugar contents, maintaining high biological yield and fresh weight, and producing favorable morphological traits, including the highest tiller number. While the 75% treatment had the lowest nitrate content, its growth performance was slightly inferior to the 50% treatment. The study clarified that a 50% mixture optimizes soil conditions to enhance both yield and quality, providing a theoretical and technical basis for rational vermicompost application in protected vegetable cultivation, in line with eco-friendly agricultural practices. This result provides theoretical support for the application of vermicompost in allium vegetable cultivation and suggests new ideas for the resource utilization of organic waste and the advancement of ecological agriculture.

However, this study has limitations. The pot experiment conditions differ from the field environments (e.g., light intensity, soil microbial communities), so field verification is needed. Another key issue is variation within the study sample. Although we employed ANCOVA was applied to control for some differences, residual, unobserved high inter-individual variation may have contributed to nonsignificant results or wide effect sizes. This suggests that future research employing repeated measures within individuals or multilevel models may better elucidate such variation.

## Data Availability

The original contributions presented in the study are included in the article/supplementary material. Further inquiries can be directed to the corresponding author.
