# Correction: Optimizing vermicompost-soil ratios for synergistic enhancement of *Allium fistulosum* growth dynamics and phytochemical quality

**DOI:** 10.3389/fpls.2025.1735464

**Published:** 2025-12-08

**Authors:** Shuangmei Gong, Xiulong Ou

**Affiliations:** Research Center for Preparation and Properties of New Functional Materials, Hanjiang Normal University, Shiyan, Hubei, China

**Keywords:** vermicompost, *Allium fistulosum*, optimal ratio, vitamin C, soluble sugar, nitrate

There was an error on page 15, in Section **5.1.7**, as published, “with VC (r=–0.43) and biological yield (r=–0.59; Table 2-12-1)”, the name of the table here is wrongly referenced. The corrected appears below.

“with VC (r=–0.43) and biological yield (r=–0.59; Table 24)”

There was an error in the caption of **Table 17** as published, “Nitrate content in *Allium fistulosum* (µgiu-1)”. The unit at the end of the title is incorrect. The corrected caption of **Table 17** appears below.

**Table 17:** Nitrate content in *Allium fistulosum* (µg· g-1).

Treatment (%Vermicompost)	Group1	Group2	Group3	Mean	±SD	±SE	95% CI
0% (Control)	200.5	250.7	166.7	190.97	21.18	12.23	(138.4, 243.6)
25%	221	234.4	192.7	216.03	21.29	12.29	(163.1, 268.9)
50%	302.1	273.4	280.2	285.23	12.28	7.09	(254.3, 316.2)
75%	127.6	179.7	122.4	143.23	31.72	18.31	(63.6, 222.9)
100%	273.4	268.2	243.2	261.6	16.15	9.32	(221.5, 301.7)

“Nitrate content in *Allium fistulosum* (µg· g^-1^)”.

There was an error in the caption of **Table 35** as published, “Soluble sugar content in *Allium fistulosum* (µgi g-1)”. The unit at the end of the title is incorrect. The corrected caption of **Table 35** appears below.

**Table 35:** Soluble sugar content in *Allium fistulosum* (µg· g-1).

Treatment (%Vermicompost)	Group1	Group2	Group3	Mean	±SD	±SE	95% CI
0% (Control)	254.1	273.5	302.2	276.6	24.31	14.04	[216.19,337.01]
25%	287.2	348.2	326.6	320.67	31.07	17.94	[243.49,397.85]
50%	379.8	423.5	417.9	407.07	22.11	12.76	[326.17,451.97]
75%	297.4	309.6	369.5	325.5	36.07	20.82	[205.92,445.08]
100%	278.5	300.8	297.1	292.13	11.42	6.59	[263.75,320.51]

“Soluble sugar content in *Allium fistulosum* (µg· g^-1^)”.

There was an error in the caption of **Figure 4** as published. In **Figure 4**, the title on the left side was erroneously in Chinese. It should be in English according to the journal’s policy. The corrected **Figure 4** appears below.

**Figure 4:**
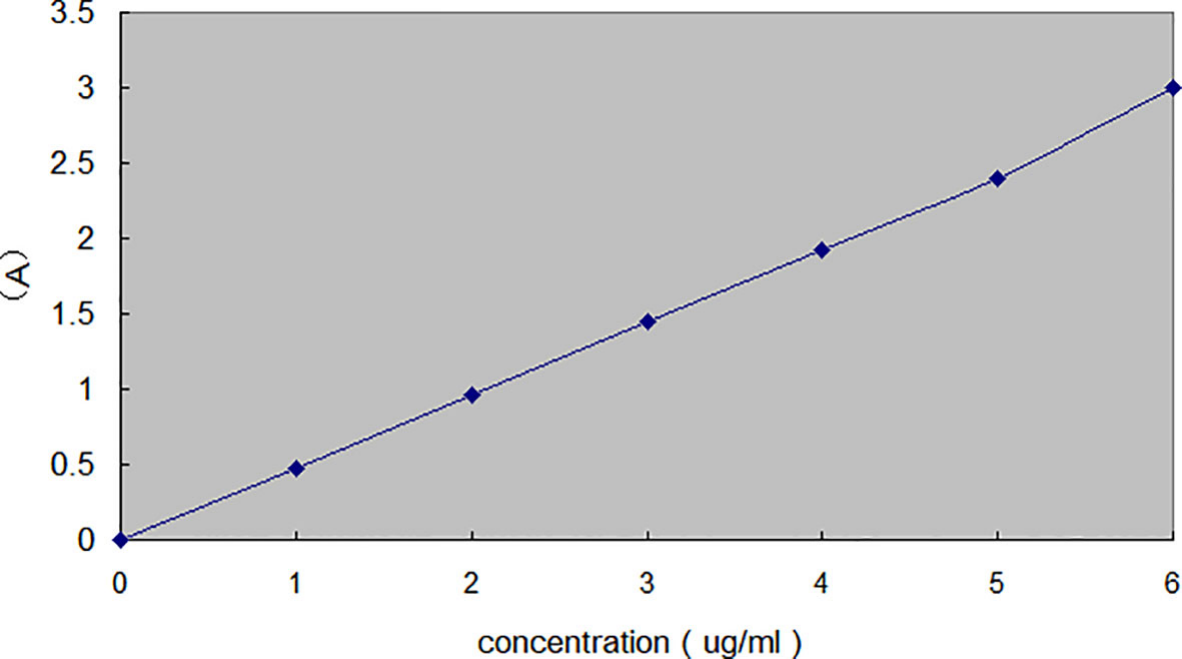
Standard curve of nitrate.

The original version of this article has been updated.

